# A Comparison of the External and Internal Demands Imposed during Conditioning Training and Match-Play in Semi-Professional and Development Female Netball Players

**DOI:** 10.3390/sports10010012

**Published:** 2022-01-10

**Authors:** Tandia G. Wood, Aaron T. Scanlan, Geoffrey M. Minett, Vincent G. Kelly

**Affiliations:** 1 School of Exercise and Nutrition Sciences, Queensland University of Technology, Brisbane, QLD 4000, Australia; geoffrey.minett@qut.edu.au (G.M.M.); v6.kelly@qut.edu.au (V.G.K.); 2 School of Health, Medical, and Applied Sciences, Central Queensland University, Rockhampton, QLD 4870, Australia; a.scanlan@cqu.edu.au

**Keywords:** team sport, PlayerLoad, load, RPE, women, intensity

## Abstract

This case series, team-based study aimed to compare the demands imposed during conditioning training and match-play in netball players. Female netball players competing at semi-professional (*n* = 9, age: 22.2 ± 3.8 years) and development (*n* = 9, age: 22.3 ± 2.9 years) levels had their internal (rating of perceived exertion (RPE)) and external (relative PlayerLoad (PL) in total and in the forwards, sideways, and vertical vectors) loads measured during conditioning-based training sessions and matches in a season. Demand variables were compared between conditioning and match-play across all players and according to position in each playing level. Conditioning training imposed higher relative PL in total and in each vector compared to match-play in semi-professional and development players (small to large effects). In contrast, RPE was significantly (*p* = 0.006) higher during match-play than conditioning training in semi-professional and development players (medium effects). Furthermore, according to playing position, significantly higher relative PL variables were evident during conditioning training than match-play in wing attack and goalkeeper semi-professional players and in goal attack, goal shooter, goal keeper, and goal defence development players. These results suggest conditioning training practices elicit adequate external intensities but inadequate internal intensities relative to match-play across positions in semi-professional and development netball players.

## 1. Introduction

Sporting performance is multi-factorial and requires a combination of fitness attributes that are unique to each sport [1]. To optimize the development of essential fitness attributes in each sport, training must be specific and stress the internal and external demands engaged during competition [1,2]. External demands encompass the physical stimuli encountered during training and match-play, while internal demands represent players’ perceptual and physiological reactions to the imposed physical stimuli [3]. In this regard, netball is a high-intensity, intermittent sport that stresses a range of different fitness attributes in players [4,5,6]. Consequently, netball strength and conditioning coaches and sports scientists must develop conditioning programs that best develop these fitness attributes and optimally prepare players for the external and internal demands of match-play [7,8]. 

Furthering the concept of training specificity in netball, the match-specific demands experienced by each playing position and playing level must be considered in developing netball-specific conditioning programs. In this way, the varied court restrictions and roles specific to each playing position in netball impose unique internal [7,9,10] and external [7,9,10,11,12,13,14] match demands across positions. For example, goal positions (goal shooters (GS) and goal keepers (GK)) experience the lowest external demands, centers (C) experience the highest external demands, and the remaining positions (wing attackers [WA], wing defenders (WD), goal attackers (GA), and goal defenders (GD)) experience external demands between these positions during match-play for female netball players [7,9,13]. In contrast, inconsistent variations between playing positions have been observed for internal demand variables (i.e., rating of perceived exertion (RPE) and heart rate (HR) responses) during netball match-play [7,9,10,13]. Furthermore, positional differences in external and internal match demands were shown to vary according to playing level when amateur domestic, under-19 years representative, and over-19 years representative female netball players were directly compared [9]. Indeed, female netball players competing at higher levels (i.e., Australian state competition) undergo greater external match demands than players competing at lower levels (i.e., Australian recreational competition) (relative PlayerLoad (PL): 10.0 ± 2.5 AU·min^−1^ vs. 7.0 ± 1.8 AU·min^−1^) [15], suggesting the match demands experienced in netball are specific to playing level in addition to playing position. Accordingly, the development of netball conditioning programs with respect to playing position likely needs to be specific to the playing level of the players involved. 

Despite the need to ensure conditioning practices prepare netball players for match demands according to playing position and playing level, comparisons in the demands experienced between conditioning sessions and matches have predominantly been conducted at the team level in netball research [7,10]. Specifically, Chandler et al. [7] reported comparable (*p* > 0.05) RPE and HR responses, but higher (*p* < 0.05) relative PL in total and each vector (forward, sideward, and vertical) during traditional conditioning involving interval and maximal aerobic speed (MAS) training without a ball and repeated high-intensity effort training involving repeated sprints, changes of direction, and jumping activities than match-play in collegiate female netball players (*n* = 8). Similarly, Simpson et al. [9] reported similar (*p* > 0.05) summated-heart-rate-zone (SHRZ) loads, but higher (*p* < 0.05) relative PL variables during conditioning drills involving repetitive intermittent running and sprinting in professional, female, netball players (*n* = 9). In addition to these team-level studies, some useful external data have been reported previously for training and match-play according to playing position in professional, female, netball players (*n* = 12) [11,12]. Interestingly, external demands (relative PL) during match-play was shown to be significantly greater (*p* < 0.01) than all training activities examined (position-specific technical/tactical work, ball control and passing drills, set-pieces, simulated match scenarios, and practical match-play) across positions [12]. However, there were some notable limitations in this research given that training demands were not provided specifically for conditioning-focused training activity, and playing positions were grouped broadly (i.e., defenders, midcourters, and goalers) rather than individually for each position [11,12]. 

Consequently, while an increasing body of literature is being established quantifying the training and match demands in female netball players, no research has compared the external and internal demands encountered during conditioning and match-play according to playing position and playing level. Such data could demonstrate how the demands imposed during conditioning practices currently compare to match-play in netball teams. Therefore, this study aimed to compare the external and internal demands encountered during netball conditioning training and match-play in semi-professional and development female netball players.

## 2. Materials and Methods

A convenience sample of 18 female netball players from the same Queensland State League Championship club participated in this study. Players were categorized into one of two playing levels being semi-professional (*n* = 9, age: 22.2 ± 3.8 years, height: 179.0 ± 6.5 cm, body mass: 71.7 ± 6.6 kg, playing experience: 4.3 ± 1.8 years, Yo-Yo Intermittent Recovery Test Level 1 (IRT1) decimal level: 16.3 ± 1.2) and development (*n* = 9, age: 22.3 ± 2.9 years, height: 176.0 ± 6.7 cm, body mass: 68.2 ± 6.6 kg, playing experience: 3.4 ± 1.4 years, Yo-Yo IRT1 level: 16.3 ± 1.5). One player for each position was analysed in each level, except for C and WA, where two players were analysed in each level. Semi-professional players were competing in a higher standard of competition than development players, with development players aiming to reach the semi-professional level. Players from both playing levels trained together; however completed skills training elements separately and competed in different compeitions. All players were free from injury throughout the study period and participated in on-court team training sessions for ~5.5 h per week across the season. Players were categorised into their playing positions by technical coaches and occupied these positions in training sessions and matches across the season. If a player occupied an alternate position in any match, that sample was included as the alternate position for that match in subsequent analyses. All procedures were approved by the University Human Research Ethics Committee (reference: 1900000783), with written and verbal informed consent acquired from each player before any data were collected.

This observational study adopted a case series, team-based approach to data collection during an entire season encompassing the pre-season (12 weeks) and an in-season phase (17 weeks) of the 2019 Sapphire Series competition. The semi-professional competition consisted of 16 matches, with 13 matches played at the same home venue and three at away venues. The development competition consisted of 15 matches, with 12 played at the same home venue and three at away venues. Players from both playing levels trained twice per week during the pre-season phase and trained twice while also competing in one competitive match per week during the in-season phase. A total of 406 data training data samples and 144 match data samples were collected in semi-professional players, while a total of 406 data training samples and 162 match data samples were collected in development players.

External demands were measured using microsensors (OptimEye X4, Catapult Innovations, Melbourne, Australia) containing tri-axial accelerometers sampling at 1000 Hz. The devices were placed between the scapulae in a neoprene vest (Catapult Innovations, Melbourne, Australia) fitted to each player prior to each training session and match. The accelerometers within these devices have been previously demonstrated to possess moderate to high reliability (CV = 4.2–14.8%) [16], and each player used the same device across all sessions to avoid any inter-device variability in measurements. Relative PL (AU·min^−1^) in total, and relative PL in each of the three vectors (forwards, sideways, and vertical) were measured using the microsensors. Only data recorded when players were on the court were included in the analysis; periods when players were not actively involved were removed from training sessions (i.e., when rested during drills or during inter-drill breaks) and matches (i.e., during substitutions and inter-period breaks). Internal demands were measured using RPE, which the same performance coach collected following the conditioning component in each training session and following each match using a modified Category Ratio-10 scale within 30 min of session completion [16]. Players reported their RPE individually to avoid peer influence [17]. 

Each training session consisted of a warm-up and a conditioning component with all players combined (i.e., semi-professional and development players together), a skill component according to each playing level (i.e., semi-professional and development players separately), and a cool-down with all players combined. The conditioning component in each training session involved high-intensity interval running using individualized MAS for each player and multiplied by the desired intensity of the session as dictated by the coach (e.g., 110% of MAS). The conditioning component of training lasted 13.4 ± 7.1 min on average per session and focused on developing aerobic capacity using straight-line running, change-of-direction drills, and netball-specific weaving drills. Only the conditioning component of each training session was included in the analyses. During the in-season phase, matches for both playing levels were 60 min in duration with a 5-min break between halves and 3-min breaks between the first and second as well as the third and fourth quarters. Two tactical time outs lasting up to 50 s each were allowed for each team per quarter. Players were interchanged during matches as decided by coaching staff, with an unlimited number of interchanges permitted.

Following each training session and match, data were downloaded from OpenField software (Catapult Innovations, Melbourne, Australia) and exported to Microsoft Excel (v15.0; Microsoft Corporation; Redmond, WA, USA) for processing. As data were not normally distributed, all variables are presented as mean ± standard deviation with median and interquartile ranges also provided. The Statistical Package for the Social Sciences (IBM SPSS v.22, Chicago, IL, USA) and Microsoft Excel (v15.0; Microsoft Corporation; Redmond, WA, USA) were used for all statistical analyses. Separate linear mixed models were conducted to assess differences between conditioning and match demand variables in each playing level (i.e., separate analyses for semi-professional and development players) with individual players set as a random effect. Further linear mixed models were used to evaluate differences between conditioning and match demand variables according to playing position separately within each playing level. Accordingly, playing position was set as a fixed effect (GS, GA, WA, C, WD, GD, and GK) and player was set as a random effect (*n* = 9) with separate models implemented for each playing level. Statistical significance was accepted at *p* < 0.05. Standardized mean differences via Cohen’s r were calculated to quantify the magnitude of difference in all pairwise comparisons and were interpreted as trivial (<0.10), small (0.10–0.29), medium (0.30–0.49), and large (>0.50) [18]. Models were plotted using GraphPad Prism (version 8.4.3, GraphPad Software, San Diego, CA, USA). 

## 3. Results

The descriptive data indicative of conditioning training and match-play demands and comparison statistics are shown in Table 1 for semi-professional netball players and Table 2 for development players. In all semi-professional players combined, analyses revealed all external demand variables were larger (*p* > 0.05) during conditioning training compared to match-play (r = 0.10–0.29, small), whereas RPE was significantly (*p* = 0.006) higher (r = 0.38, medium) during match-play than conditioning training (Table 1). Comparisons in all development netball players combined revealed conditioning training imposed significantly (*p* < 0.01) higher (r = 0.24–0.26, small) relative PL in total and each vector than match-play, with RPE significantly (*p* = 0.001) higher (r = 0.34, medium) during match-play than conditioning training.

**Figure 1:**
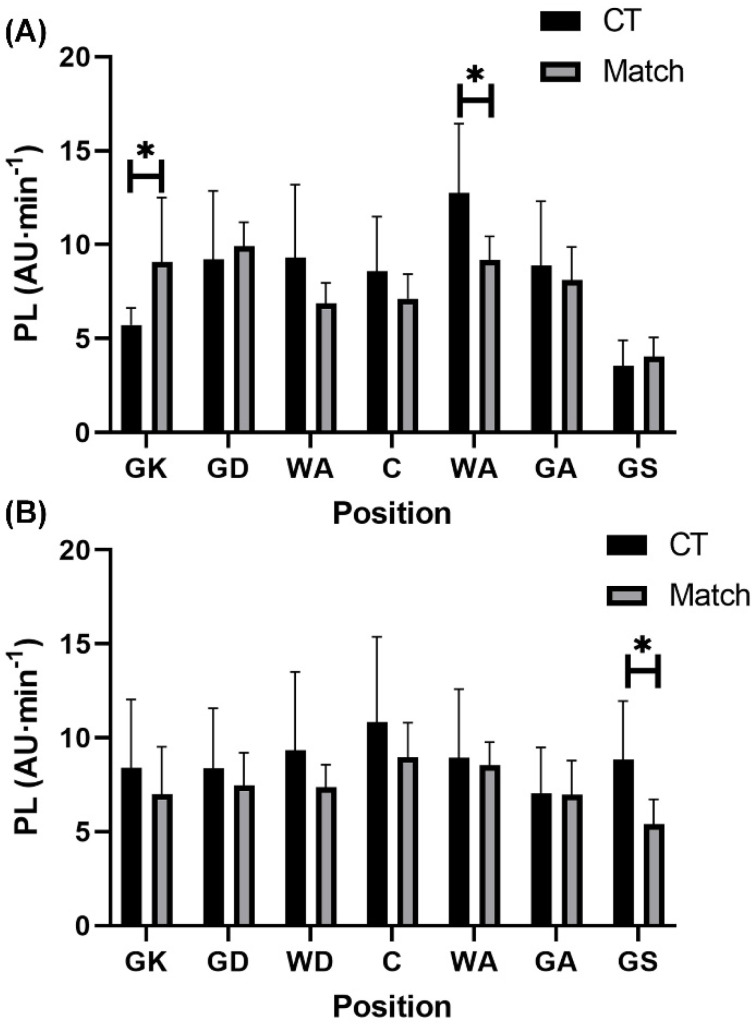
Positional comparisons in relative total PlayerLoad (mean ± standard deviation) between conditioning training and match-play in (**A**) semi-professional and (**B**) development female netball players. Note: * denotes significant difference between conditioning training and match-play (*p* < 0.05). Abbreviations: PL = PlayerLoad; AU = arbitrary units; CT = conditioning training; GK = goal keeper; GD = goal defence; WD = wing defence; C = center; WA = wing attack; GA = goal attack; GS = goal shooter.

**Figure 2:**
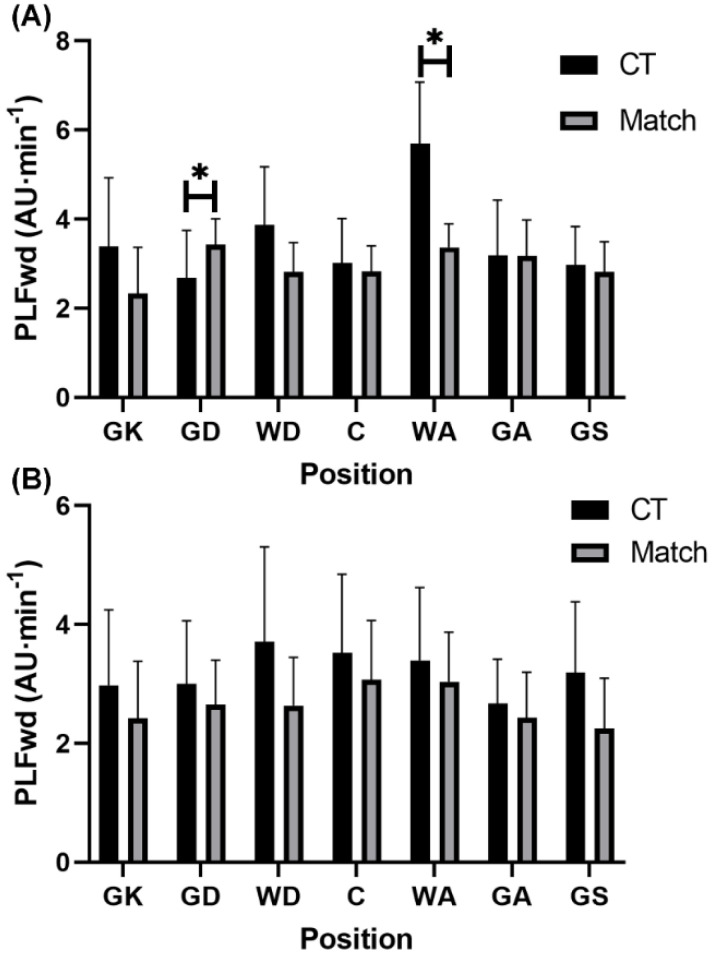
Positional comparisons in relative total PlayerLoad in the forwards vector (mean ± standard deviation) between conditioning training and match-play in (**A**) semi-professional and (**B**) development female netball players. Note: * denotes significant difference between conditioning training and match-play (*p* < 0.05). Abbreviations: PLFwd = PlayerLoad in the forwards vector; AU = arbitrary units; CT = conditioning training; GK = goal keeper; GD = goal defence; WD = wing defence; C = center; WA = wing attack; GA = goal attack; GS = goal shooter.

**Figure 3:**
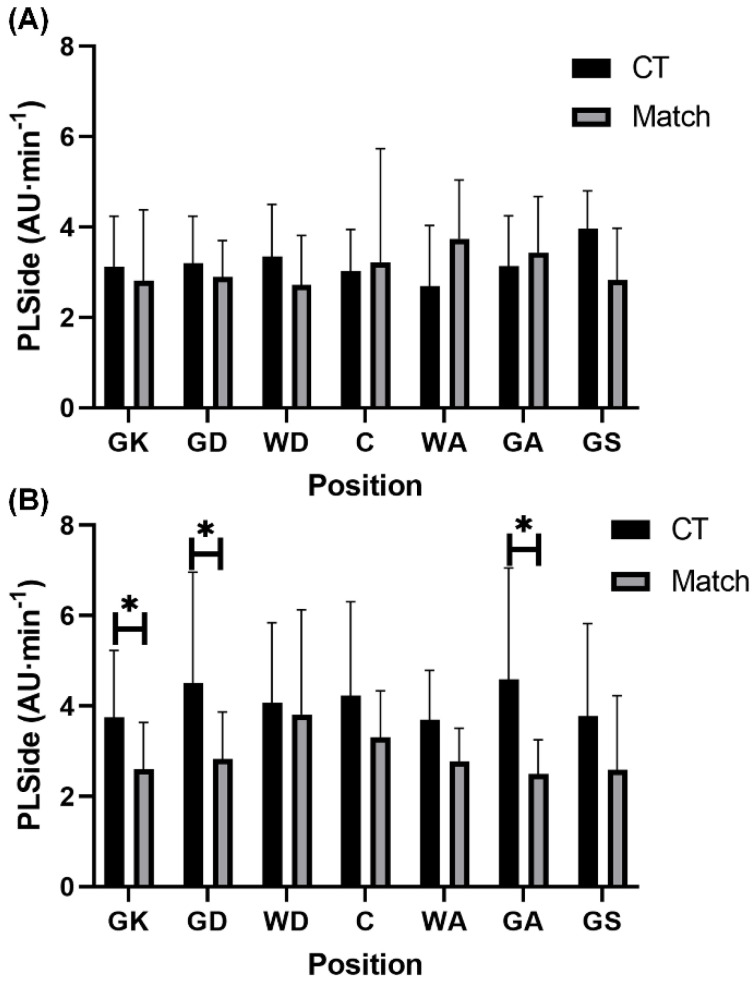
Positional comparisons in relative total PlayerLoad in the sideways vector (mean ± standard deviation) between conditioning training and match-play in (**A**) semi-professional and (**B**) development female netball players. Note: * denotes significant difference between conditioning training and match-play (*p* < 0.05). Abbreviations: PLSide = PlayerLoad in the sideways vector; AU = arbitrary units; CT = conditioning training; GK = goal keeper; GD = goal defence; WD = wing defence; C = center; WA = wing attack; GA = goal attack; GS = goal shooter.

**Figure 4:**
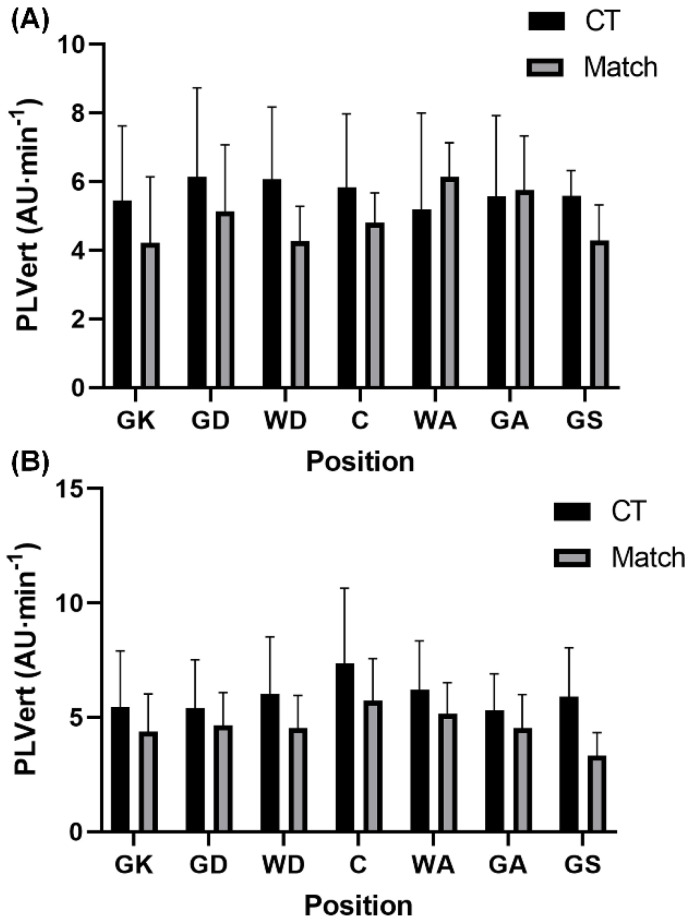
Positional comparisons in relative total PlayerLoad in the vertcial vector (mean ± standard deviation) between conditioning training and match-play in (**A)** semi-professional and (**B**) development female netball players. Abbreviations: PLVert = PlayerLoad in the vertical vector; AU = arbitrary units; CT = conditioning training; GK = goal keeper; GD = goal defence; WD = wing defence; C = center; WA = wing attack; GA = goal attack; GS = goal shooter.

**Figure 5:**
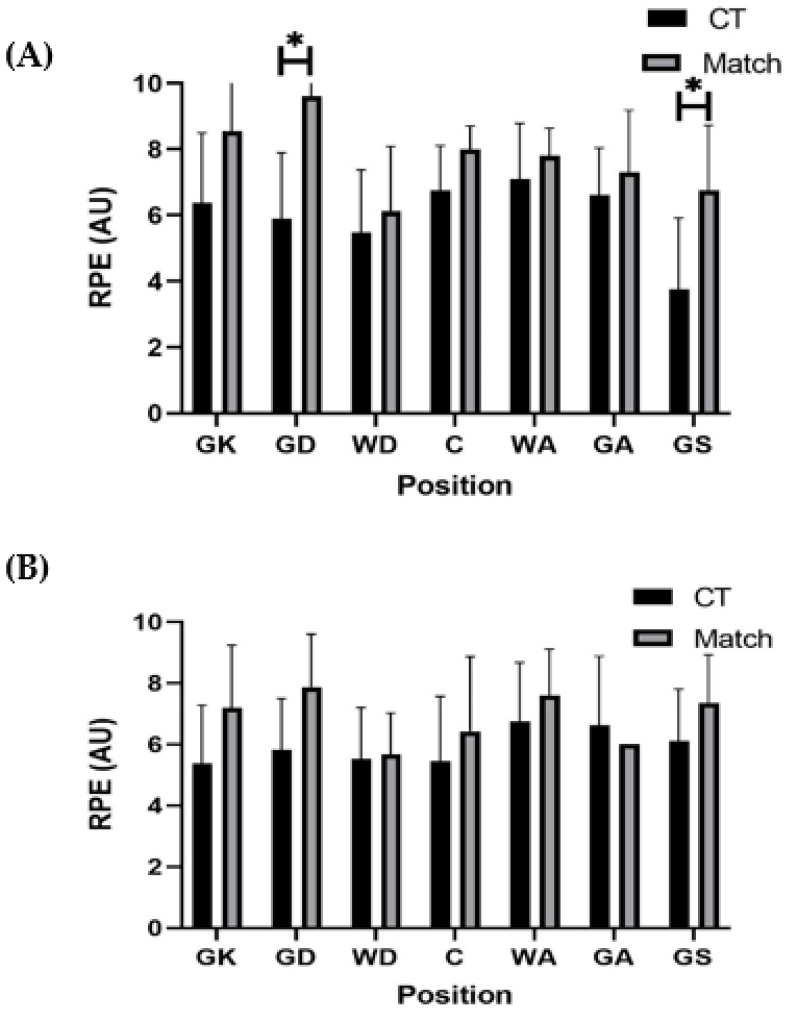
Positional comparisons in rating of perceived exertion (mean ± standard deviation) between conditioning training and match-play in (**A**) semi-professional and (**B**) development female netball players. Note: * denotes significant difference between conditioning training and match-play (*p* < 0.05). Abbreviations: RPE = rating of perceived exertion; AU = arbitrary units; CT = conditioning training; GK = goal keeper; GD = goal defence; WD = wing defence; C = center; WA = wing attack; GA = goal attack; GS = goal shooter.

Comparisons between conditioning and match-play according to playing level and position in each demand variable are shown in Figure 1, Figure 2, Figure 3, Figure 4 and Figure 5. For relative total PL, GK (9.10 ± 3.43 AU·min^−1^ vs. 5.70 ± 0.89 AU·min^−1^; *p* = 0.03, r = 0.56, large) and WA (12.75 ± 3.70 AU·min^−1^ vs. 9.17 ± 1.27 AU·min^−1^; *p* = 0.001, r = 0.54, large) both encountered significantly higher demands during conditioning compared to match-play among semi-professional players (Figure 1). Among development players, GS experienced a significantly higher (*p* = 0.01, r = 0.53, large) relative total PL during conditioning training (8.54 ± 3.26 AU·min^−1^) compared to match-play (5.41 ± 1.33 AU·min^−1^) (Figure 1).

For relative PL in the forwards vector, GD encountered a significantly lower (*p* = 0.001, r = 0.40, medium) demands during conditioning training (3.32 ± 1.46 AU·min^−1^) compared to match-play (2.65 ± 0.89 AU·min^−1^) among semi-professional players (Figure 2). To the contrary, WA encountered a significantly higher (*p* = 0.001, r = 0.45, medium) relative PL in the forwards vector during conditioning training (4.42 ± 1.40 AU·min^−1^) compared to match-play (3.37 ± 0.53 AU·min^−1^) in semi-professional players (Figure 2). 

For relative PL in the sideways vector, GA (5.30 ± 6.03 AU·min^−1^ vs. 2.50 ± 0.75 AU·min^−1^; *p* = 0.002, r = 0.31, small), GD (5.62 ± 5.62 AU·min^−1^ vs. 2.91 ± 1.53 AU·min^−1^; *p* = 0.001, r = 0.31, small), and GK (5.46 ± 6.64 AU·min^−1^ vs. 2.58 ± 1.02 AU·min^−1^; *p* = 0.003, r = 0.29, small) each encountered significantly higher relative PL in the sideways vector during conditioning training than match-play among development players (Figure 3). While for RPE, both GD (5.90 ± 1.99 AU vs. 9.60 ± 0.55 AU; *p* = 0.02, r = 0.79, large) and GS (3.75 ± 2.17 AU vs. 6.75 ± 1.98 AU; *p* = 0.04, r = 0.59, large) experienced significantly higher responses during match-play compared to conditioning training among semi-professional players (Figure 5). All other positional comparisons in each variable between conditioning and match demands were non-significant (*p* > 0.05) and trivial to small in magnitude across both playing levels (Figure 1, Figure 2, Figure 3, Figure 4 and Figure 5). 

## 4. Discussion

This study aimed to compare the external and internal demands between conditioning training and match-play according to playing position in semi-professional and development female netball players. The present findings revealed that relative PL in total and each vector were higher (small effects) during conditioning compared to match-play in semi-professional (*p* > 0.05) and development (*p* < 0.05) players. In contrast, RPE was higher (*p* < 0.05, medium effects) during match-play than conditioning in semi-professional and development players. Furthermore, selected positions across both semi-professional (WA and GK) and development (GA, GD, GK, and GS) players also experienced greater (*p* < 0.05, small to large effects) external demands during conditioning training than match-play, while selected positions in semi-professional players (GD and GS) experienced greater RPE during conditioning than match-play (*p* < 0.05, large effects). These findings suggest that relative external demands of conditioning training are greater than those encountered during match-play, particularly in specific positions, while the perceptual demands of conditioning training fail to meet those experienced during match-play in semi-professional and development players.

The higher relative external demands evident during conditioning training compared to match-play across semi-professional and development female netball players in the present study aligns with existing data provided for female netball players [7,10]. Specifically, Simpson et al. [10] reported higher (*p* < 0.05) relative PL in total and each vector during conditioning training involving similar intermittent running and sprinting drills to those monitored in the present study in professional female netball players, while Chandler et al. [7] observed higher (*p* < 0.05) relative PL in total and each vector during conditioning predicated on interval and MAS training or repeated, multi-directional, high-intensity efforts in collegiate female netball players. Although the relative PL values in total (13.1–18.5 AU·min^−1^ vs. 7.1–7.3 AU·min^−1^) and in each vector (4.8–12.8 AU·min^−1^ vs. 2.6–7.7 AU·min^−1^) were considerably higher during conditioning training in past research examining professional [10] and collegiate [7] female netball players than the present study, the consistent trends across studies suggest netball teams may be adequately prepared for the external demands of match-play through the typical conditioning approaches adopted in practice across various playing levels. Indeed, it appears that with similar intermittent endurance capacities (Yo-Yo IRT1 decimal level: semi-professional = 16.3 ± 1.2; development = 16.3 ± 1.5), prescription of conditioning drills based on individualized MAS appear to yield consistent external loads, above those experienced during match-play, irrespective of playing level in netball players. Given external demands (relative PL) during match-play have been shown to significantly exceed (*p* < 0.01) those experienced in other training drills (position-specific technical/tactical work, ball control and passing drills, set-pieces, simulated match scenarios, and practical match-play) in professional female netball players across playing positions [12], the present data support the need for conditioning-oriented training tasks to ensure players are exposed to adequate external stimuli in preparation for match demands.

While previous research has identified that match demands vary according to playing position across various playing levels (i.e., recreational to professional) in netball players [7,9,10,13,14], no research has investigated direct differences between conditioning training and match-play demands according to playing position. The current investigation showed that conditioning training imposed significantly greater external demands specifically in GK (relative total PL) and WA (relative PL in total and in the sideways vector) positions at the semi-professional level. Traditionally, the GK position experiences the lowest external demands and the WA position experiences intermediate external demands relative to other playing positions during netball match-play [7,10,13]. In the semi-professional team, both the GK and WA positions had high fitness levels relative to international-standard female netball players [19]. In turn, each player’s conditioning training was potentially tailored to their individualized aerobic capacity, which may have elicited high external loads that were not reflected in match-play at the semi-professional level, especially in the GK and WA positions. Conversely, significantly lower relative PL in the forwards vector was evident during conditioning training compared to matches in the GD position at the semi-professional level. This finding may be due to the defensive activities readily performed in the GD position, whereby off-ball guarding has been identified as the most demanding activity performed during match-play in professional female netball players [20] with this defensive activity being absent from conditioning drills. At the development level, significantly higher relative PL in the sideways vector in GA, GD, and GK positions, and significantly higher relative total PL in the GS position were evident during conditioning compared to match-play. Notably, these positions are goal-based, limiting their involvement across all plays and their ability to cover as much distance because of the positional court restrictions during matches. Comparatively, the conditioning drills (e.g., straight-line MAS running) were characterised by fewer movement restrictions, allowing goal-based players to reach higher external intensities in various directions than they would customarily achieve during matches [7]. 

In addition to external demand variables, analysis of internal demand variables showed RPE was significantly higher for match-play than conditioning training for both semi-professional and development players. Thus, the augmented perceptual intensities during match-play may be attributed to various factors related to the competitive environment accompanying official matches. In this regard, competitive basketball matches are postulated to increase cognitive and somatic anxiety due to the opposition faced, game location, crowds, and importance of winning [21], which might have also played a role in elevating player RPE during match-play in the present study. Moreover, position-specific findings suggest GD and GS semi-professional players may be particularly prone to mismatches in RPE between conditioning and match-play, possibly due to the criticality of these positions during scoring scenarios and match outcomes. Specifically, previous data in provincial-level female netball players suggest GS has less ability to peak under pressure and cope with adversities and GD experience higher levels of worry than other positions during matches [22], which may elevate RPE. In contrast to the present results, previous research examining collegiate female netball players found identical mean RPE values (5 AU) between conditioning involving interval and MAS training (5 AU), conditioning involving repeated, multidirectional, high-intensity efforts (5 AU), and match-play (5 AU) using the same RPE scale [7]. Given that similar RPE values were observed during conditioning (6.12 AU vs. 5.85 AU) and higher RPE values were apparent during match-play (7.73 AU vs. 7.17 AU) in the present study compared to previous research [7], a possible reason for variations in findings between studies may be due to differences in match contexts and activities being encountered across the monitored teams. For instance, the semi-professional and development competitions examined in the present study likely evoked different competitive environments than the collegiate competition examined previously [7], including variations in match locations, outcomes, and score-lines, which have been shown to induce small-moderate effects on RPE in semi-professional, male basketball players [23]. Moreover, existing data suggest changes of direction (r = 0.79), jumps (r = 0.76), and accelerations/decelerations (r = 0.75) most strongly correlate with perceptual demands during netball training and match-play in professional female netball players [24]. In turn, netball matches played at high playing levels imposes a frequent rate of these activities [10], with higher level players possessing superior jump heights [4] and netball-specific multi-directional movement speed [25] than lower level players, possibly further underpinning the greater RPE during match-play observed in the present study than in collegiate female netball players reported previously [7]. 

The present research provides important insight regarding the external and internal demands of conditioning and match-play in semi-professional and developmental netball players, yet it is not without limitations. Firstly, while the comparison in demands between playing levels was a novel component of the present study, only one team from each level was recruited. Consequently, the findings are likely representative of team-specific approaches rather than the wider netball population, so the inclusion of more teams is encouraged in future research to provide a more robust reflection of conditioning training and match demands according to playing position in female netball players. Similarly, only one to two players per position were analysed. However, given the case series, team-based approach to this research and its explorative nature, these findings provide a foundation for further research across a wider assortment of netball teams and players. Secondly, although several PL variables were collected with the available monitoring technology in the present study, inertial variables (i.e., accelerations, decelerations, changes of direction, and jumps) were not able to be measured for deeper insight into player demands. Furthermore, considering internal load, objective measurement approaches such as heart rate monitoring were unavailable, but would provide further insight into the demands experienced by netball players according to playing level and position. Finally, various drills employed in the conditioning component of training sessions were grouped collectively in analyses. Consequently, a more thorough investigation of conditioning training with specific drills analysed separately (e.g., running drills, small-sided games under various formats) is recommended for a wider understanding of the demands associated with different conditioning approaches relative to match demands in netball players.

## 5. Conclusions

The present study demonstrates that higher external intensities are experienced during conditioning training than match-play in netball players, particularly in WA and GK positions at the semi-professional level and goal-based positions at the development level. In contrast, RPE was higher during match-play than conditioning across both playing levels, particularly in the semi-professional players’ GD and GS positions. These outcomes suggest that players may be physically prepared for the external intensities reached during matches across positions, as external conditioning demands were comparable or higher; however, they may need further stimuli imposed during conditioning to mimic the perceptual intensities encountered during matches across positions. In turn, coaches may incorporate conditioning tasks with increased cognitive load or match-like pressure to elevate perceptual intensities combined with intense physical demands indicative of match-play. 

## Figures and Tables

**Table 1:** Descriptive data and comparison statistics between conditioning training and match-play demands in semi-professional female netball players.

Variable	Conditioning Training		Match-Play		Comparison Statistics
	Mean ± SD	Median (IQR)	Mean ± SD	Median (IQR)	*p*-Value, Cohen’s r, Effect Magnitude
Relative PL (AU·min^−1^)	8.92 ± 3.54	9.11 (5.80–11.50)	7.12 ± 2.31	7.12 (5.43–8.98)	0.075, 0.29, small
Relative PL forwards (AU·min^−1^)	3.32 ± 1.46	3.20 (2.20–4.29)	2.65 ± 0.89	2.74 (1.92–3.37)	0.204, 0.27, small
Relative PL sideways (AU·min^−1^)	3.15 ± 1.15	3.17 (2.30–3.97)	2.89 ± 1.38	2.80 (2.15–3.48)	0.566, 0.10, small
Relative PL vertical (AU·min^−1^)	5.79 ± 2.43	5.73 (3.81–7.41)	4.67 ± 1.70	4.49 (3.34–6.01)	0.208, 0.26, small
RPE (AU)	6.12 ± 1.94	6.00 (5.00–7.00)	7.73 ± 1.98	8.00 (7.00–9.00)	0.006, 0.38, medium

Abbreviations: SD = standard deviation; IQR = interquartile range; PL = PlayerLoad; AU = arbitrary units.

**Table 2:** Descriptive data and comparison statistics between conditioning training and match-play demands in development female netball players.

Variable	Conditioning Training		Match-Play		Comparison Statistics
	Mean ± SD	Median (IQR)	Mean ± SD	Median (IQR)	*p*-Value, Cohen’s r, Effect Magnitude
Relative PL (AU·min^−1^)	8.75 ± 3.82	8.40 (5.43–11.30)	7.28 ± 2.14	7.63 (5.28–8.91)	0.002, 0.24, small
Relative PL forwards (AU·min^−1^)	3.15 ± 1.40	3.01 (2.05–4.17)	2.55 ± 0.91	2.56 (1.79–3.26)	0.009, 0.26, small
Relative PL sideways (AU·min^−1^)	5.65 ± 3.68	3.73 (2.45–11.53)	2.91 ± 1.50	2.75 (1.91–3.34)	0.001, 0.26, small
Relative PL vertical (AU·min^−1^)	5.82 ± 2.67	4.72 (3.19–5.85)	4.52 ± 2.67	5.55 (3.58–7.67)	0.002, 0.24, small
RPE (AU)	5.85 ± 1.87	6.00 (5.00–7.00)	7.17 ± 1.82	8.00 (6.00–9.00)	0.001, 0.34, medium

Abbreviations: SD = standard deviation; IQR = interquartile range; PL = PlayerLoad; AU = arbitrary units.

## Data Availability

The data is not yet publicly available.

## References

[1] Reilly T., Morris T., Whyte G. (2009). The specificity of training prescription and physiological assessment: A review. J. Sports Sci..

[2] Campbell P., Peake J., Minett G. (2018). The specificity of rugby union training sessions in preparation for match demands. Int. J. Sports Physiol. Perform..

[3] Fox J., O’Grady C., Scanlan A. (2020). The relationships between external and internal workloads during basketball training and games. Int. J. Sports Physiol. Perform..

[4] Simpson M., Jenkins D., Leveritt M., Kelly V. (2019). Physical profiles of elite, sub-elite, regional and age-group netballers. J. Sports Sci..

[5] Sirotic A., Coutts A. (2007). Physiological and performance test correlates of prolonged, high-intensity, intermittent running performance in moderately trained women team sport athletes. J. Strength Cond. Res..

[6] Thomas C., Comfort P., Jones P., Dos’ Santos T. (2017). Strength and conditioning for netball: A needs analysis and training recommendations. Strength Cond. J..

[7] Chandler P., Pinder S., Curran J., Gabbett T. (2014). Physical demands of training and competition in collegiate netball players. J. Strength Cond. Res..

[8] McKeown I., Chapman D., Taylor K., Ball N. (2016). Time course of improvements in power characteristics in elite development netball players entering a full-time training program. J. Strength Cond. Res..

[9] King D., Cummins C., Hume P., Clark T. (2020). Physical demands of amateur domestic and representative netball in one season in New Zealand assessed using heart rate and movement analysis. J. Strength Cond. Res..

[10] Simpson M., Jenkins D., Kelly V. (2020). Workload differences between training drills and competition in elite netball. Int. J. Sports Physiol. Perform..

[11] Brooks E., Benson A., Fox A., Bruce L. (2020). Physical movement demands of elite-level netball match-play as measured by an indoor positioning system. J. Sports Sci..

[12] Brooks E., Benson A., Fox A., Bruce L. (2021). Movement intensity demadns between training activities and competition for elite female netballers. PLoS ONE.

[13] Van Gogh M., Wallace L., Coutts A. (2020). Positional demands and physical activity profiles of netball. J. Strength Cond. Res..

[14] Young C., Gastin P., Sanders N., Mackey L., Dwyer D. (2016). Player load in elite netball: Match, training, and positional comparisons. Int. J. Sports Physiol. Perform..

[15] Cormack S., Smith R., Mooney M., Young W., O’Brien B. (2014). Accelerometer load as a measure of activity profile in different standards of netball match play. Int. J. Sports Physiol. Perform..

[16] Barrett S., Midgley A., Lovell R. (2014). PlayerLoad™: Reliability, convergent validity, and influence of unit position during treadmill running. Int. J. Sports Physiol. Perform..

[17] Minett M., Fels-Camilleri V., Bon J., Impellizzeri F., Borg D. (2021). Peer presence increases session ratings of perceived exertion. Int. J. Sports Physiol. Perform..

[18] Cohen J. (1992). A power primer. Psychol. Bull..

[19] Tanner R.K., Gore C.J. (2013). Physiological Tests for Elite Athletes.

[20] Bailey J., Gastin P., Mackey L., Dwyer D. (2017). The player load associated with typical activities in elite netball. Int. J. Sports Physiol. Perform..

[21] Williams M., Dalbo V., Fox J., O’Grady C., Scanlan A. (2021). Comparing weekly training and game demands according to playing position in a semiprofessional basketball team. Int. J. Sports Physiol. Perform..

[22] Grobbelaar H., Eloff M. (2011). Psychological skills of provincial netball players in different playing positions. S. Afr. J. Sport Phys. Educ. Res..

[23] Fox J., Stanton R., Sargent C., Scanlan A. (2020). The impact of contextual factors on game demands in starting, semiprofessional, male basketball players. Int. J. Sports Physiol. Perform..

[24] Simpson M., Jenkins D., Scanlan A., Kelly V. (2020). Relationships between external-and internal-workload variables in an elite female netball team and between playing positions. Int. J. Sports Physiol. Perform..

[25] Scanlan A., Peralta P., Gass G., Mungovan S. (2018). Discriminative validity of a novel, high-intensity, netball-specific circuit test in elite female netball players. S. Afr. J. Sport Phys. Educ. Res..

